# Claudin-containing exosomes in the peripheral circulation of women with ovarian cancer

**DOI:** 10.1186/1471-2407-9-244

**Published:** 2009-07-20

**Authors:** Jianghong Li, Cheryl A Sherman-Baust, Miyun Tsai-Turton, Robert E Bristow, Richard B Roden, Patrice J Morin

**Affiliations:** 1 Laboratory of Cellular and Molecular Biology, National Institute on Aging, Baltimore, MD, USA; 2 Department of Pathology, The Johns Hopkins Medical Institutions, Baltimore, MD, USA; 3 Department of Oncology, The Johns Hopkins Medical Institutions, Baltimore, MD, USA; 4 Department of Gynecology & Obstetrics, The Johns Hopkins Medical Institutions, Baltimore, MD, USA

## Abstract

**Background:**

The absence of highly sensitive and specific serum biomarkers makes mass screening for ovarian cancer impossible. The claudin proteins are frequently overexpressed in ovarian cancers, but their potential as prognostic, diagnostic, or detection markers remains unclear. Here, we have explored the possible use of these proteins as screening biomarkers for ovarian cancer detection.

**Methods:**

Claudin protein shedding from cells was examined by immunoblotting of conditioned culture media. The presence of claudins in exosomes released from ovarian cancer cells was demonstrated by sucrose gradient separation and immunogold electron microscopy experiments. Claudin-4-containing exosomes in the plasma of ovarian cancer patients were evaluated in a pilot panel of 63 ovarian cancer patients and 50 healthy volunteers. The CA125 marker was also assessed in these samples and compared with claudin-4 positivity.

**Results:**

We show that full-length claudins can be shed from ovarian cancer cells in culture and found in the media as part of small lipid vesicles known as exosomes. Moreover, 32 of 63 plasma samples from ovarian cancer patients exhibited the presence of claudin-4-containing exosomes. In contrast, only one of 50 samples from individuals without cancer exhibited claudin-4-positive exosomes. In our small panel, at a specificity of 98%, the claudin-4 and CA125 tests had sensitivities of 51% and 71%, respectively. The two tests did not appear to be independent and were strongly correlated.

**Conclusion:**

Our work shows for the first time that claudin-4 can be released from ovarian cancer cells and can be detected in the peripheral circulation of ovarian cancer patients. The development of sensitive assays for the detection of claudin-4 in blood will be crucial in determining whether this approach can be useful, alone or in combination with other screening methods, for the detection of ovarian cancer.

## Background

Ovarian cancer is the fifth cause of cancer deaths in women in the United States [1]. However, when detected early, ovarian cancer has an excellent prognosis, with a 5-year survival rate exceeding 90%. Unfortunately, because of a lack of obvious symptoms and the current limitations in detection techniques, only a small number of ovarian cancers are detected early, while the vast majority (70%) is diagnosed as advanced disease when the survival rate is approximately 30% [1]. Among the problems related to ovarian cancer detection is the lack of highly specific and sensitive serum biomarkers. The most clinically useful ovarian cancer biomarker, CA125, has been used to assess response to treatment and monitor recurrence of CA125-positive tumors [2], but unfortunately lacks both sensitivity and specificity required for the efficient detection of ovarian cancer in the general population [3,4]. The sensitivity of CA125 is limited by the fact that a significant proportion of ovarian cancers do not express this marker, especially tumors of clear cell, undifferentiated, and mucinous histological subtypes [5]. In addition, multiple gynecological conditions can lead to elevated CA125 levels, reducing its overall specificity [4,6]. A combination of approaches involving serum CA125 detection and ultrasound imaging, while promising, still yielded positive predictive values of approximately 20% for ovarian cancer detection [7-9]. It has been suggested that combining multiple serum markers may help achieve the sensitivity and specificity required for the screening of ovarian cancer [4,10]. There has therefore been a significant interest in the identification and development of new ovarian cancer markers.

Claudins are a family of transmembrane proteins crucial in the formation and function of tight junctions, the most apical contact between polarized cells [11]. Several claudin genes have been found aberrantly expressed in cancer [12]. In particular, we and others have shown that claudin-3 and claudin-4 are elevated in ovarian cancer [13-22]. The functional consequences of overexpressed claudin-3 and -4 in cancer remain unclear, but these proteins may be important for invasion, motility, survival, and metastasis [23-31]. In addition, certain claudins have been suggested as prognostic markers in various cancers. For example, in ovarian cancer, claudin-3 and claudin-7 expression has been shown to be inversely correlated with survival [21,22]. Claudin-3 and claudin-4 expression was shown to be associated with poor clinical outcome in endometrial cancer [32] and renal cell carcinoma [33].

Exosomes are small (40–100 nm) membrane vesicles of endocytic origins shed by multiple cell types, such as lymphocytes, dendritic cells, neurons, intestinal cells, and others [34]. Interestingly, tumor cells have been shown to release increased amounts of exosomes [35] and, in fact, these vesicular structures were first observed in the peripheral circulation of women with ovarian cancer [36]. Although the exact mechanistic details of exosome formation remain unclear, the protein composition of exosomes reflects the cell of origin and may include both cytoplasmic and membrane proteins. For example, exosomes from antigen-presenting cells contain MHC class I molecules [37], and exosomes from enterocytes contain intestinal enzymes [38]. It has therefore been hypothesized that tumor-derived exosomes may contain specific biomarkers for the detection of various cancers [39].

In this report, we show for the first time that intact claudin proteins are released from ovarian cancer cells. The released claudins are incorporated into exosomes and can be detected in the media of cells expressing claudins, as well as in the peripheral circulation of a majority of women with ovarian cancer. Isolation of blood exosomes followed by the detection of claudins may represent a novel approach for the detection of ovarian cancer.

## Methods

### Cell Culture, Preparation of Cell Lysate and Culture Media

Ovarian cancer cell lines OVCAR-2, OVCAR-3, OVCA420, OVCA433, BG-1, Hey, and UCI101 were cultured in McCoy's 5A medium (Invitrogen Life Technologies, Carlsbad, CA), supplemented with 10% fetal bovine serum (FBS) and antibiotics (100 units/ml penicillin and 100 μg/ml streptomycin). The origin of these cell lines has been documented [40]. Colon cancer cell lines HCT-116 and SW-480, and the breast cancer cell line MCF-7 were also cultured in McCoy's 5A medium. Ovarian cancer cell line A2780 was maintained in RPMI-1640 medium supplemented with 10% FBS, antibiotics (100 units/ml penicillin and 100 μg/ml streptomycin), and 4 μg/ml bovine insulin (Invitrogen Life Technologies).

Whole cell lysates were prepared by adding lysis buffer (62.5 mM Tris-HCl (pH 6.8), 10% glycerol, 2% SDS) to 80% confluent cell cultures. Protein concentration was determined using the BCA assay kit (Pierce, Rockford, IL). For the preparation of the conditioned culture media, cells were grown to 80% confluency and then with FBS-free media for 24 hours. The conditioned culture media were collected and centrifuged at 1,000 rpm for 10 min to remove cells and debris, and were further concentrated by Centriplus YM-10 (Millipore) prior to immunoblotting.

### Immunoblotting

Fifteen μg of lysate proteins or 10 μl of concentrated culture media were separated by 10–20% SDS-PAGE (Tris-Glycine gels, Invitrogen Life Technologies, Carlsbad, CA), and transferred to PVDF membranes (Millipore, Bedford, MA). The membranes were blocked with 5% nonfat dry milk, washed in Tris-Buffered Saline with 0.1% Tween 20 buffer and probed with primary antibodies against claudin-1 (1:200 dilution), claudin-3 (1:200 dilution), claudin-4 (1:400 dilution), or claudin-5 (1:200 dilution). All the primary antibodies were obtained from Invitrogen. After washing in TBST, membranes were incubated with HRP-conjugated secondary antibody (anti-rabbit or anti-mouse IgG: 1:10,000; Amersham Biosciences Corp, Piscataway, NJ). For detection, enhanced chemiluminesence was carried out using the ECL plus kit (Amersham Biosciences Corp). Rabbit anti-claudin-4 antibody (Abcam Inc., Cambridge, MA) was used at a 1:200 dilution for the detection of claudin-4 from plasma exosomes.

### Exosome Preparation from Culture Media

BG-1 cells were cultured to 80–90% confluency and then incubated FBS-free media for 24 hours to allow accumulation of exosomes. The conditioned culture media were harvested and subjected to two centrifugations (2,000 g and 10,000 g) to remove cells and debris. Exosomes were then pelleted from the media at 100,000 g for 2 hour using a SW41Ti rotor, washed once in cold PBS, and resuspended in 50 μl of PBS.

### Sucrose Gradient

Fresh exosomes obtained by ultracentrifugation were resuspended in 2 ml of 2.5 M sucrose, 20 mM Tris-HCl (pH 7.4), loaded at the bottom of a 8 ml linear sucrose gradient (2.0 – 0.25 M sucrose, 20 mM Tris-HCl (pH 7.4)) in a SW41 Ti tube, and centrifuged at 270,000 g for 16 hour at 4°C. Gradient fractions (1 ml) were collected from the bottom of the tube and the presence of claudins in the various fractions was assessed by immunoblotting as described above. The density of each fraction was assessed by measuring the weight of 500 μl aliquots.

### Transmission Electron Microcopy (TEM)

Exosomes isolated from BG-1 culture media were adsorbed to glow discharged 400 mesh carbon-coated parlodion copper grids (Pella) for 2 min, and briefly rinsed in 1× filtered PBS (Gibco). Exosomes on grids were then fixed in 1% glutaraldehyde and negatively stained with 1% phosphotungstic acid, before TEM. For immunogold electron microscopy, the exosomes on the grids were blocked in filtered 1% BSA for 10 min and the grids floated in the primary antibody solution (1:2 dilution of claudin-4 in blocking solution) in a humidity chamber for 2 hours at room temperature. The primary antibody was omitted from grids used as negative controls. After primary antibody incubation, grids were washed in blocking solution for 10 min, briefly rinsed in PBS, and incubated with the secondary 6-nm gold-conjugated goat anti-rabbit antibody (Jackson Immunochemical; diluted 1:40 in PBS) for 1 hour in a humidity box. The samples were then placed on PBS for 10 min., fixed in 2% glutaraldehyde for 5 min, rinsed again in PBS, and then deionized water. Finally the grids were floated on 2 consecutive drops of filtered aqueous 2% Uranyl Acetate for 1 min each (Pella) and blot dried with Whatman # 1 filter paper. Both negatively stained and immunogold labeled grids were viewed on a Hitachi H-7600 TEM operating at 80 kV and images were captured with a 1 K × 1 K CCD by AMT.

### Pulse-Chase Experiments and Immunoprecipitation

BG-1 cells were cultured to 80–90% confluency, incubated for 1 hour with methionine- and cysteine-free DMEM medium (Invitrogen) and then labeled for 20 min with 100 μCi/ml L-[^35^S]methionine and L-[^35^S]cysteine using the Protein Labeling Mix (PerkinElmer NEG072). After labeling, the cells were incubated with complete McCoy's 5A medium for 40 min to allow [^35^S] incorporation into proteins. After chasing in serum-free McCoy's 5A medium for 0, 2, 6, or 24 hours, the culture media were centrifuged at 2,000 g for 10 min, followed by 10,000 g for 20 min at 4°C, concentrated to 500 μl, and mixed with 2× immunoprecipitation (IP) buffer and protease inhibitor cocktail (Sigma) before IP. In order to assess total incorporation, a cell lysate was prepared immediately after labeling using cold IP buffer (50 mM Tris-Cl (pH 7.4), 150 mM NaCl, 1 mM EDTA, and 1% NP-40) containing protease inhibitor cocktail. The lysate was cleared by centrifugation at 10,000 g for 20 min at 4°C.

For immunoprecipitation, both culture media and cell lysates were incubated overnight with 10 μg claudin-4 antibody (Invitrogen) and 50 μl protein A-Sepharose beads (Amersham Biosciences) at 4°C. After washing the beads three times with IP buffer, immune complexes were boiled for 5 min in 2× sample buffer and analyzed by SDS-PAGE. Radiolabeled bands were quantitated using a phosphorimager.

### Plasma Samples and CA125 detection

Plasma was obtained from whole blood samples received from women prior to surgery for ovarian cancer at Johns Hopkins School of Medicine, as well as healthy volunteers with informed consent. All the patients used for this study were diagnosed with high-grade serous ovarian cancer. The details of the cancer diagnosis are included in Table 1. For both cancer patients and healthy volunteers, plasma CA125 levels were determined by an ELISA kit from Panomics Inc. according to the manufacturer's instructions.

**Table 1:** Cancer patient information, CA125 values, and claudin-4 positivity.

Sample	Stage	CA125 (unit/ml)	Claudin-4*
P5	4	11576	2
P41	3	641	2
P4	4	387	2
P36	4	5255	2
P34	3	213	2
P32	4	950	2
P3	4	3225	2
P29	4	6084	2
P26	4	1995	2
P25	4	9937	2
P24	3	965	2
P23	3	147	2
P21	3	2169	2
P10	3	10601	2
P54	4	164	2
P56	4	96	2
P57	4	826	2
P59	4	1251	2
P63	3	3707	2
P72	3	1110	2
P78	3	1773	2
P79	3	1805	2
P82	3	456	2
P39	3	599	1
P37	3	411	1
P22	3	963	1
P52	4	1447	1
P53	4	911	1
P60	4	590	1
P67	3	726	1
P75	3	1632	1
P80	3	349	1
P9	4	95	0
P8	3	85	0
P7	4	184	0
P6	4	45	0
P40	4	55	0
P38	3	5921	0
P31	3	29	0
P30	3	451	0
P2	4	208	0
P19	4	0	0
P14	3	221	0
P1	4	0	0
P51	4	1407	0
P55	4	502	0
P58	4	744	0
P61	3	380	0
P62	3	3775	0
P64	3	120	0
P65	3	232	0
P66	3	11	0
P68	3	3890	0
P69	3	600	0
P70	3	54	0
P71	3	83	0
P73	3	115	0
P74	3	38	0
P76	3	50	0
P77	3	0	0
P81	3	101	0
P83	3	194	0
P84	3	73	0

*scoring of claudin-4 intensity was performed as follows: 2, strong band; 1, weak to moderate band; 0, undetectable.

### Isolation of circulating exosomes

Control and patient plasma (200 μl) were diluted to 1 ml with RPMI-1640 medium (Invitrogen), centrifuged at 2,000 g for 10 min at 4°C, the supernatants were then transferred to 15 ml tubes (avoiding pellet at the bottom or fat at the top if present) and further diluted to 10 ml with cold PBS, and centrifuged at 10,000 g for 20 min at 4°C to remove large debris. The samples were then transferred to ultracentrifuge tubes and exosomes were pelleted at 100,000 g for 2 hour using SW41Ti rotor. Following centrifugation, exosomes were resuspended in 50 μl PBS. Ten μl of exosomes were used for immunoblotting and Abcam anti-claudin-4 antibody was used to detect claudin-4 from plasma exosomes.

### Statistics

The relationship between claudin-4 and CA125 positivity was evaluated using Fisher's Exact test.

## Results

### Cancer cells in culture release claudin proteins

**Figure 1:**
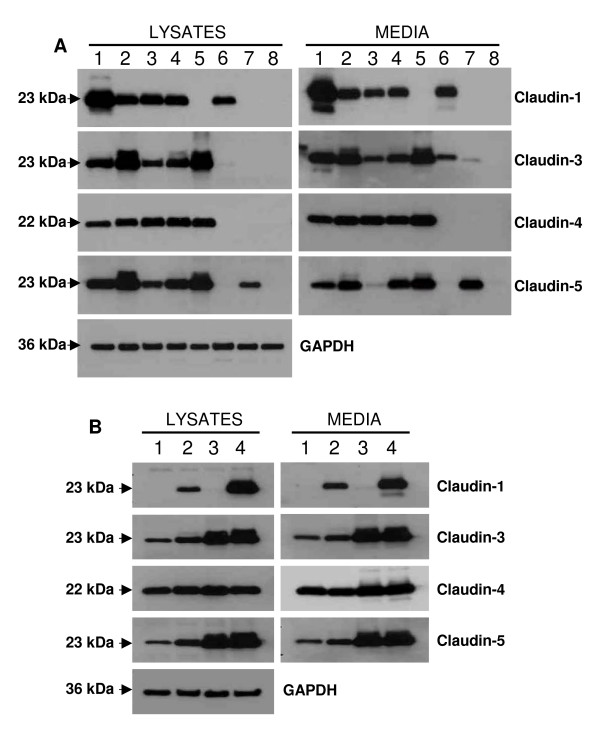
**Claudin proteins are shed from cancer cell lines**. *A*, Cell lysates and culture media were collected from ovarian cancer cell lines, and analyzed by immunoblotting for the expression of claudin-1, claudin-3, claudin-4 and claudin-5. Ovarian cancer cell lines used: OVCAR-2 (1), OVCAR-3 (2), OVCA420 (3), OVCA433 (4), BG-1 (5), Hey (6), UCI101 (7), and A2780 (8). *B*, Cell lysates and culture media were collected from various cancer cell lines, and analyzed by immunoblotting for the expression of claudin-1, claudin-3, claudin-4 and claudin-5. Cell lines analyzed are: colon cancer HCT-116 (1), colon cancer SW-480 (2), breast cancer MCF-7 (3), and ovarian cancer OVCAR-3 (4). Claudins expressed in the various cell lines were released in the media.

In order to test the possibility that claudin proteins may be released from cells, we looked for the presence of claudins in conditioned culture media from ovarian cancer cell lines. Culture media from cell lines OVCAR-2, OVCAR-3, OVCA420, OVCA433, BG-1, Hey, UCI101, and A2780 were collected and immunoblotting was performed to detect claudin-1, claudin-3, claudin-4, and claudin-5 in the conditioned media (Figure 1A). Interestingly, the full-length claudins were detected in the media of several ovarian cancer cell lines. The presence of claudins in the media corresponded to expression of these claudins in the various cell lines and claudin proteins were not detected in the media of cell lines lacking their expression. The correlation between expression in the lysates and presence in the media was striking, with the exception of claudin-3 in HEY cells, where expression of the protein was very low in the lysate, but claudin-3 was nonetheless detectable in the media.

Because the expression of claudins have been detected in other cancers [12], we next sought to determine whether these proteins might also be secreted by other cancer cell lines. Two colon cancer lines (SW-480 and HCT-116) and one breast cancer cell line (MCF-7) were examined for the release of claudins. Again, we found that these cell lines shed claudin proteins in the media at levels roughly proportional to their expression patterns (Figure 1B). Overall, our findings show that, when expressed in a cell, a fraction of the claudin proteins can be released in the media.

### The released claudin proteins are incorporated into exosomes

**Figure 2:**
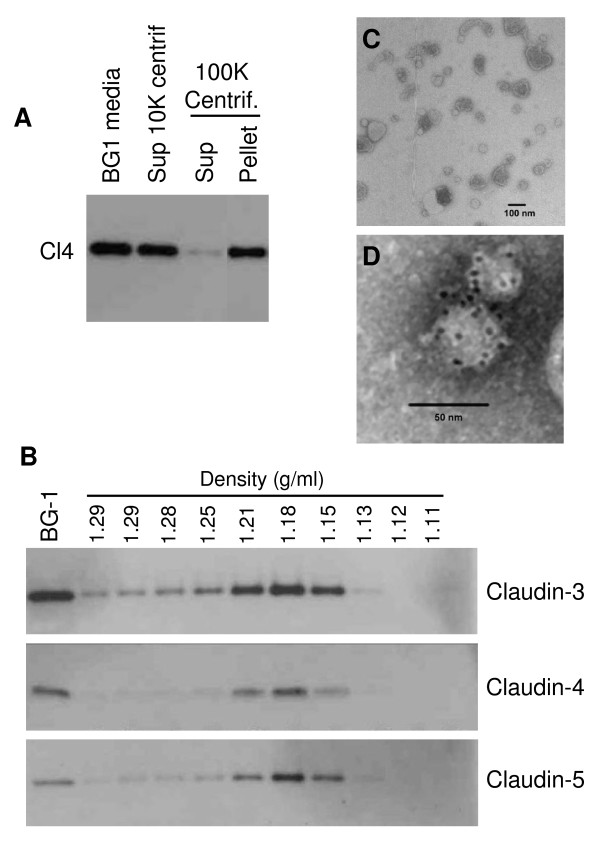
**Released claudin proteins are present in exosome particles**. *A*, BG-1 culture media was centrifuged sequentially and analyzed by immunoblotting for the expression of claudin-4. Claudin-4 was sedimented by a 100,000 g centrifugation (100 K Centrif.) but not by 10,000 g centrifugation (10 K Centrif.). *B*, Sucrose gradient analysis of the 100,000 g pellet. Following separation of the pellet on a sucrose gradient, ten successive fractions of increasing densities were collected and analyzed by immunoblotting for the expression of the indicated claudin proteins. The density of the various fractions was measured and is indicated on top of the lanes. *C*, Transmission electron microscopy image of exosomes released from ovarian cancer cells. Exosomes isolated from BG-1 culture media by the 100,000 g centrifugation were fixed, negatively stained, and observed by TEM. The exosomes observed are 40–100 nm in diameter and exhibit the typical cup-shaped morphology. *D*, Indirect immunogold labeling with anti-claudin-4 antibody followed by a secondary antibody coupled to 6-nm gold particles. The gold particles (dark dots on the figure) clearly localize to the exosomes.

To determine whether claudins are released in the media as free molecules or as part of larger complexes, we focused on claudin-4 and performed a series of sedimentation experiments using media from the BG-1 ovarian cancer cell line. BG-1 cells were chosen as a model for these experiments as they shed relatively high levels of claudin-4. Low speed centrifugation (10,000 g) removed cells and cell debris from the media, but did not significantly affect the amount of claudin-4 in the supernatant (Figure 2A). In contrast, high speed ultracentrifugation (100,000 g) of the media significantly reduced the amount of claudin-4 detectable in this fraction and transferred this protein to the pellet. These results suggested that a significant portion of the released claudin was part of a relatively large complex that could be sedimented at 100,000 g. Because claudins are membrane proteins, we considered the possibility that they may be released within lipid vesicles known as exosomes. To test this hypothesis, the pellet from the BG-1 media was resuspended in PBS and subjected to a sucrose gradient separation. Fractions of increasing densities were collected and assayed by immunoblotting for the presence of claudin-4. The bulk of the claudin signal was identified in fractions of densities of 1.15–1.21 g/ml (Figure 2B), corresponding to the reported densities of exosomes [34,41].

In order to further demonstrate that the released claudin proteins were contained within exosomes, electron microscopy experiments were performed. When examined by TEM using negative staining, the pellet from BG-1 media contained a large number of cup-shaped vesicles of approximately 40–100 nm in diameter (Figure 2C), consistent with the reported morphology and size of exosomes [34]. Immunogold electron microscopy experiments with an anti-claudin-4 antibody confirmed the presence of claudin-4 within the exosomes (Figure 2D). Negative controls lacking primary antibody did not exhibit the presence of labeled exosomes (data not shown).

**Figure 3:**
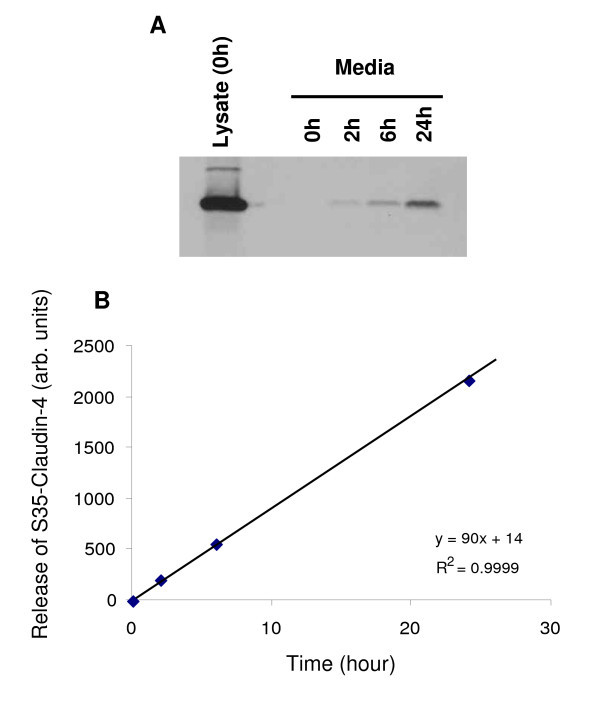
**The kinetics of claudin release**. *A*, BG-1 cells were pulsed-labeled with [^35^S]methionine and [^35^S]cysteine, chased for various times as indicated, before the media was collected. Claudin-4 was immunoprecipited from the media or from the [^35^S]-labeled whole cell lysate (as control for the total amount of labeled claudin-4) and analyzed by SDS-PAGE. Claudin-4 is released rapidly in the media, with radiolabeled claudin-4 detectable as early as 2 hours post-labeling. *B*, The radiolabeled claudin-4 released into the culture media was quantitated by phosphorimaging of the blot shown in (A) and plotted against time. Claudin-4 increase in the media is linear with time.

To gain a better understanding of the kinetics of release of claudin-containing exosomes by ovarian cells, BG-1 cells were pulse-labeled with [^35^S]methionine/[^35^S]cysteine and the media was collected at different times before immunoprecipitation with a claudin-4 antibody. In order to evaluate the total amount of labeled protein, a cell lysate was also produced immediately following [^35^S]-labeling and immunoprecipitated with the anti-claudin-4 antibody. Labeled claudin-4 was present in the media as early as 2 hours post-labeling (Figure 3A) and reached a level of approximately 3–4% of the total labeled claudin-4 after 24 hours. The kinetics of release of claudin-4 in the media were linear (Figure 3B), suggesting that the labeled claudin-4 protein was relatively stable during this timeframe.

### Claudin-4 can be found in blood exosomes from ovarian cancer patients, but not in healthy controls

**Figure 4:**
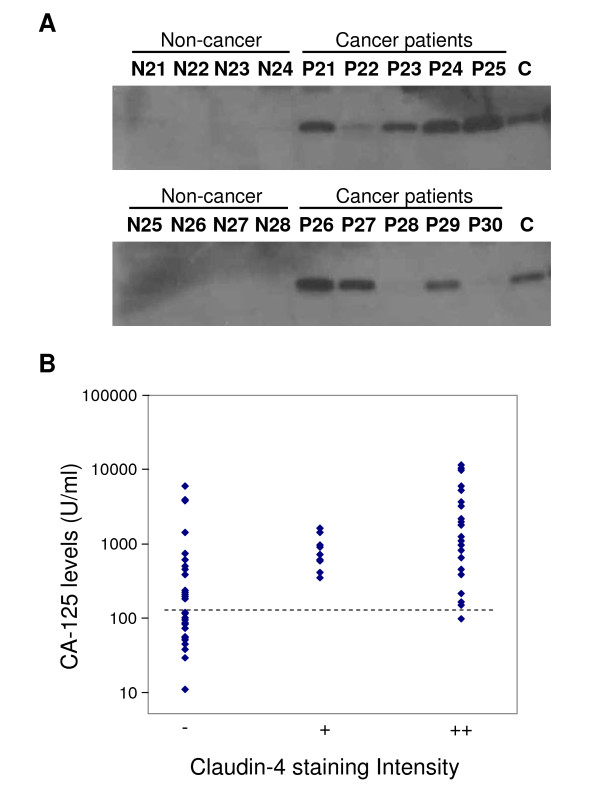
**Claudin-4 expression in the plasma of ovarian cancer patients**. *A*, Representative examples claudin-4 detection in plasma samples by immunoblotting. N21 to N28 are non-cancer plasma controls. P21 to P30 are plasma from ovarian cancer patients. C is a positive control (BG-1 culture media). *B*, CA125 levels in plasma of ovarian cancer patients are compared to claudin-4 expression levels. ++, claudin-4 level is greater than 0.15 μl of BG-1 24 h culture media; +, claudin-4 level is greater than 0.07 μl of BG-1 24 h culture media; -, no detectable expression of claudin-4. CA125 is plotted on a log scale and the threshold of 125 U/ml is shown by a dotted line.

Our finding that claudins can be released from ovarian cancer cells suggests that these proteins may become useful for the detection of ovarian cancer in patients. We first sought to determine whether exosome-associated claudins could indeed be detected in the peripheral circulation of ovarian cancer patients. Plasma samples from 63 high-grade serous ovarian cancer patients treated at Johns Hopkins University, as well as 50 healthy volunteers were assessed. Exosomes were purified from the plasma by ultracentrifugation and assayed for the presence of claudin-4 and claudin-3 by immunoblotting. Several plasma samples from patients with ovarian cancer exhibited high levels of claudin-4, while blood from control individuals showed very low or undetectable levels (Figure 4A). The pattern obtained with claudin-3 was somewhat similar, but the background levels in the plasma of unaffected women were much higher (data not shown). Claudin-3 was therefore considered less likely than claudin-4 to represent a useful plasma-based ovarian cancer biomarker and was not studied further. Among the 63 ovarian cancer patients, 23 samples demonstrated a strong claudin-4 band with 9 additional samples exhibiting low to moderate levels (Table 1). Thirty one samples had no detectable claudin-4 protein. In contrast, among the control group (n = 50), only one sample was positive (at a low level) and all the other plasma samples were negative (Table 2). Based on these figures, claudin-4 has a sensitivity of 51% (32/63) and specificity of 98% (49/50) for the detection of ovarian cancer.

**Table 2:** CA125 levels and claudin-4 positivity in the cancer-free controls

Sample	CA125 (unit/ml)	Claudin-4*
N32	0	1
N2	0	0
N3	41	0
N4	4	0
N5	122	0
N6	44	0
N7	0	0
N8	0	0
N9	0	0
N21	0	0
N22	8	0
N23	0	0
N24	0	0
N25	76	0
N26	27	0
N27	6	0
N28	4	0
N29	0	0
N30	0	0
N31	20	0
N33	1	0
N34	0	0
N35	0	0
N36	47	0
N37	0	0
N38	0	0
N39	0	0
N19	9	0
N15	0	0
N11	0	0
N1	0	0
N10	30	0
N41	0	0
N42	0	0
N43	0	0
N44	22	0
N45	80	0
N46	0	0
N47	16	0
N48	99	0
N49	27	0
N51	0	0
N52	0	0
N53	85	0
N54	0	0
N55	9	0
N56	4	0
N57	0	0
N58	2	0
N59	171	0

*scoring of claudin-4 intensity was performed as follows: 2, strong band; 1, weak to moderate band; 0, undetectable.

### Comparison of claudin-4 and CA125 levels

CA125 is the most clinically useful ovarian cancer biomarker. We therefore wished to compare the claudin-4 positivity with CA125 values in our tumor panel. Tables 1 and 2 show claudin-4 and CA125 values for cancer patients and healthy controls, respectively. Cancer patients that demonstrated high levels of CA125 also tended to exhibit high levels of claudin-4 (Figure 4B). With a CA125 threshold of 125 U/ml, chosen to obtain a specificity of 98%, the CA125 sensitivity was approximately 71% (45/63). At that threshold, several of the claudin-4-negative plasma samples were in the CA125-positive range, including some samples with very high CA125 (such as P38, P62, and P68). 31 of the 32 cancer patients identified as positive by claudin-4 were also positive using the CA125 marker and 17 patients were negative for both (Table 3). Using Fisher's exact test, the association between claudin-4 and CA125 positivity was shown to be highly significant (P = 3.4 × 10^-6^), confirming the lack of independence between these two markers. The false positive obtained with CA125 (N59) was different than the one obtained by claudin-4 (N32) (Table 2).

**Table 3:** CA125 and Claudin-4 immunopositivity in ovarian cancer plasma samples

	Cl4 +	Cl4 -
CA125 Positive	31	14
CA125 Negative	1	17

## Discussion

Because of the significant difference between the survival rates of patients with early and advanced ovarian cancer, there has been a sustained interest in the identification of blood markers that may help detect ovarian cancer at an early stage. However, because of a relatively low specificity and sensitivity, the CA125 assay is currently not deemed appropriate for the screening of the general population [4]. Importantly, it has been shown that combining CA125 with other markers can improve the predictive value, leading to speculation that the development of a useful test may be possible, provided the right combination of markers [4,10]. In this context, there has been a considerable effort in trying to identify novel circulating biomarkers for ovarian cancer. Several ovarian biomarkers, such as Kallikreins, osteopontin, leptin, HE-4, LPA, MUC1 and SLPI, [3,10,18,42-45] have been identified in the past several years through various approaches, including gene expression profiling and proteomics analysis of ovarian tumors. The usefulness of these markers alone or in various combinations still remains to be determined, although initial studies using with multi-marker combinations are promising [10].

Claudin-3 and -4 have emerged as proteins commonly elevated in ovarian cancer [13-22], but because they are integral membrane proteins, they had not previously been suggested as serum markers. Here, we demonstrate that full length claudins can be shed into the media of ovarian cancer cell lines (Figure 1). Consistent with the fact that these proteins are transmembrane proteins, we found the claudins to be present in small membrane vesicles known as exosomes, rather than soluble entities in the media (Figures 2 and 3). This conclusion was drawn from several lines of evidence. First, we showed that a significant portion of the claudin signal was found in the pellet following high speed ultracentrifugation, suggesting that these proteins are indeed part of large complexes. Second, using density gradient analysis, we demonstrated that the claudin complexes exhibited a density range consistent with the density of exosomes. Finally, electron microscopy experiments clearly showed the presence of cup-shaped 40–100 nm vesicles in our preparations, and immunogold electron microscopy clearly localized claudin-4 to these exosomes (Figure 2C,D). In an attempt to clarify the kinetics of claudin shedding in ovarian cells, we performed a pulse-chase experiment and showed that claudins were released relatively rapidly from the cells following protein synthesis (a claudin band was detectable as early as 2 hours post-labeling) and that the kinetics of release were linear, even 24 hr post-labeling (Figure 3). These results further confirmed the release of newly synthesized claudin in the media, and the linearity suggested that the released claudin-4 is stable in the media.

Because tumors are known to produce exosomes and release them in the bloodstream, our findings suggested that plasma from ovarian cancer patients may exhibit elevated levels of claudin-containing exosomes. The analysis of a pilot panel of ovarian cancer cases (63 cancer patients and 50 controls) did indeed show that several ovarian cancer patients exhibited extremely high levels of claudin-4 in the plasma, while control individuals did not typically show elevated amounts of this protein (Figure 4). The current results therefore suggest that there is potential for claudins to become useful ovarian cancer detection markers either alone or in combination with other markers/strategies.

While the current work establishes that claudins can indeed be present in the peripheral circulation of ovarian cancer patients, several important issues will need to be clarified in subsequent studies before claudin-4 can be considered a viable biomarker for ovarian cancer detection. As currently performed (immunoblotting), the claudin-4 test has a lower sensitivity than CA125 (51% vs 71% at a specificity of 98%) and there was significant overlap between CA125 and claudin-4 positivity in our pilot study. In fact, of the 32 positive samples identified by the claudin-4 test, 31 were also CA125 positive and 17 samples (27%) were negative by both the CA125 and claudin-4 assays. Nonetheless, an improvement in the detection strategy (including the development of an ELISA-based test) may significantly increase the sensitivity of the claudin-4 test. Moreover, since the patients included in this study were restricted to those with late stages, high grade serous ovarian cancers, further studies with a larger cohort will be necessary to clearly assess the potential of claudin-4 in other subtypes, in low grades, and in early stages patients. Significantly, unlike CA125, claudin-4 and claudin-3 have been shown elevated in all subtypes of ovarian cancer [14]. In addition, we have identified a stage I patient that is negative for CA125, but exhibit claudin-4 positivity (data not shown), suggesting that it is possible for early CA125-negative cases to be claudin-4 positive. Because of the known limitations of CA125 for the detection of early ovarian cancer, the finding that claudin-4 can possibly detect early cases of ovarian cases is particularly intriguing. A follow up study with a more sensitive detection strategy and a larger number of ovarian cancer patients (with tumors of various stages and grades) tested for circulating claudin-4 will help to clarify these issues. The current work shows that a small proportion of unaffected individuals may be recorded as positive (1 out of 50), but a more accurate determination will have to be performed and its association with CA125 levels assessed.

Circulating tumor-derived exosomes have previously been found in cancer patients [36,46,47] and the detection of blood exosomes has therefore been suggested as a strategy for the detection of cancer [39,46]. However, to fully exploit a detection assay based on exosomes, it is crucial to distinguish tumor-derived exosomes from those derived from normal cells. It has been shown that the ovarian cancer proteins CD24 and EpCAM can indeed be found in exosomes from ascites fluid of ovarian cancer patients [47]. In addition, circulating exosomes from ovarian cancer patients were shown to contain a specific set of microRNAs corresponding to the expression profiles identified in the tumors from the corresponding patients [46]. The data presented here show that ovarian cancer-derived exosomes contain high levels of the membrane protein claudin-4. While claudins were not detectable directly from plasma, the enrichment of exosomes from the plasma (through ultracentrifugation) allowed us to detect these proteins with great sensitivity. This finding, together with the reports of other ovarian markers in exosomes [46,47], suggests that exosomes may contain several proteins that may also be useful as markers, and the ease of exosomes enrichment provides an elegant approach for the purification of these proteins from other plasma components. Exosomes derived from different cancers will likely have a different protein composition [48], suggesting a possibility to identify a cancer based on the composition of the detected exosomes. For example, following the isolation of plasma exosomes from a patient, the analysis of the exosome composition may help determine whether the exosomes are derived from normal or tumor cells. The detection of abnormal exosomes would suggest the presence of a tumor in the patient and the exact composition of these exosomes may then allow the identification of the site of origin of the tumor.

## Conclusion

Much work remains to be done to clearly demonstrate a clinical use for claudins and exosomes in the detection of ovarian cancer. In particular, the development of a high-throughput approach for the detection of circulating claudin-4 will be crucial, as our current approach involving exosomes purification and detection by immunoblotting does not allow for large scale population testing and does not appears to provide improved sensitivity over the currently used CA125 marker. However, our work shows for the first time that claudins may indeed become useful as ovarian cancer detection biomarkers because of their incorporation into exosomes shed by ovarian cancer cells. Whether the information provided by a blood-based claudin-4 test will be complementary to CA125 or merely redundant remains to be fully assessed. In any case, because there is evidence that most cancer cells release exosomes, the use of exosomes-associated biomarkers represents an exciting new possibility for the detection of a variety of cancers.

## Competing interests

The authors declare that they have no competing interests.

## Authors' contributions

PJM is the project leader and directed the study. He is the corresponding author. JL was the main investigator of the study, performed the experiments, and assisted in writing the draft and subsequent version of the manuscript. CASB participated in the microcopy experiments and discussion of the results. MTT performed the CA125 ELISA. MTT, REB, and RBR were in involved in patient sample collection and the Johns Hopkins ovarian cancer tumor bank. RBR helped in planning experiments and subsequent interpretation and discussion. All authors reviewed and approved the final manuscript.

## Pre-publication history

The pre-publication history for this paper can be accessed here:

http://www.biomedcentral.com/1471-2407/9/244/prepub
